# Exploring final-year nursing students’ experiences in dysphagia management at an Eastern Cape University

**DOI:** 10.4102/sajcd.v72i2.1135

**Published:** 2025-11-27

**Authors:** Amanda Shusha, Siya C. Zingaye, Esona Phundulu, Awonke Ngqina, Refiloe V. Masinge, Khomotjo S. Kgare

**Affiliations:** 1 Department of Natural and Rehabilitation Sciences, Faculty of Health Sciences, University of Fort Hare, East London, South Africa

**Keywords:** dysphagia, undergraduate training, interprofessional education, multidisciplinary approach, nursing students

## Abstract

**Background:**

A multidisciplinary team approach is crucial for effectively assessing and managing dysphagia. With nurses as key players in healthcare, there is limited knowledge about nursing students’ experiences and exposure to dysphagia as part of their undergraduate training.

**Objectives:**

To explore the experiences of final-year nursing students at a university in the Eastern Cape regarding dysphagia management.

**Method:**

A qualitative descriptive phenomenological approach was employed. Semi-structured interviews were conducted with 12 final-year nursing students who had prior exposure to dysphagia management. Thematic analysis was used to analyse and identify key themes in the data.

**Results:**

Four main themes emerged: (1) confidence and knowledge, (2) clinical exposure, (3) current dysphagia management practices and (4) development of clinical skills. The participants expressed varying levels of theoretical knowledge and clinical exposure regarding dysphagia management, with the majority reporting that the focus in their lectures was on the insertion of a nasogastric tube as part of dysphagia management. Exposure to dysphagia depended on which ward the students were placed in during the clinical training. There was notably limited knowledge among participants on the role of speech therapists in dysphagia management.

**Conclusion:**

The expressed knowledge gaps and limited clinical exposure hindered nursing students’ confidence and competence in dysphagia management.

**Contribution:**

This study highlights the importance of reevaluating the way dysphagia is taught to nursing students. By providing more theoretical and practical exposure and integrating interprofessional education, nursing students’ knowledge, confidence and practice in dysphagia can be improved.

## Introduction and literature review

Dysphagia, or difficulty swallowing, is a serious, life-threatening clinical issue that impacts people of all ages and health backgrounds. Dysphagia emanates from anatomical, neuromuscular, infectious and inflammatory diseases, with conditions like stroke being the most dominant aetiology. Consequences of untreated dysphagia include malnutrition, dehydration, aspiration pneumonia and a significantly diminished quality of life for those affected and even death (Abu-Sneineh & Saleh, 2018; Bremare et al., 2016b; Dallal-York & Troche, 2024; Jayes et al., 2024; Nilsen et al., 2022; Robbertse & De Beer, 2020a). Given the pivotal role of nurses in patient care, understanding nursing students’ knowledge and preparedness regarding dysphagia is essential, as they represent the future workforce responsible for early recognition and intervention (Adams & Coutts, 2025; Chen et al., 2024; Jones & Porterfield, 2020b). Because of its complexity, dysphagia management requires a team approach involving multiple healthcare professionals, including nurses, speech-language pathologists (SLPs), gastroenterologists, radiologists, otolaryngologists and neurologists (Rudd et al., 2024; Oliveira et al., 2022). According to the World Health Organization (WHO), collaboration among healthcare disciplines leads to better coordination of health services, appropriate referrals to specialists, improved health outcomes for patients with chronic conditions and enhanced overall patient safety and care (Brooks et al., 2016).

This study examines the experiences of final-year nursing students at an Eastern Cape University regarding dysphagia management, with a specific focus on their learning environment. The article will concentrate on the roles of nurses in relation to the students’ academic and clinical training.

Nurses are described as the epicentre of key personnel in the healthcare system because of their role in the provision and coordination of care (Oldland et al., 2020). As a result of their professional mandate or scope of practice, their role in dysphagia is unquestionable because they are, in most cases, the first professionals to observe and identify eating and drinking difficulties in patients and are in constant contact with patients (Jung et al., 2022; Seedat & Strime, 2021a; South African Nursing Council, 2019). Their responsibilities may include initial screening, monitoring of symptoms and implementing treatment plans in collaboration with SLPs (Rivelsrud et al., 2024). According to international literature, the screening can be completed within 24 h of admission for patients with acute neurological impairment, and it can significantly improve clinical outcomes (Oliveira et al., 2022). Additionally, this early screening within 24 h of admission can significantly reduce chest infections, inpatient deaths and time spent nil by mouth (Hines et al., 2014). Effective nursing interventions in dysphagia management can enhance early recognition, timely referrals to SLPs and overall patient care (Hines et al., 2010, 2014).

Considering the importance of the nurses’ role in dysphagia identification and management, research into their training and knowledge in feeding and swallowing disorders is gaining momentum. Research indicates that nursing students often feel unprepared and lack confidence in this area (Olímpio et al., 2024). A study conducted in South Africa by Seedat and Strime (2021b) also reported similar findings in that not all participants (nurses) in their study were confident or well-equipped to work with dysphagia. Moreover, the younger and recently qualified nurses were found to be lacking practical experience, and the participants felt that their undergraduate training did not adequately prepare them for working with dysphagic patients (Ebrahimi et al., 2012). Cichero et al. (2017b) discovered notable differences in the knowledge levels of nursing students concerning dysphagia management. Because of the nurses’ limited undergraduate and in-service training in dysphagia, they may be unable to identify symptoms, complications or manage dysphagia effectively, affecting their overall practices (Knight et al., 2020). These gaps may compromise patient care and raise ethical and medico-legal concerns, particularly when nurses are not equipped to identify or manage swallowing difficulties effectively (Seedat & Strime, 2021c). On the patients’ end, the consequences include aspiration of ingested foods and liquids, resulting in pneumonia (Feng et al., 2019). These results of knowledge gaps and lack of practical experience in nursing students and qualified nurses raise concern, particularly in low- and middle-income countries (LMICs) such as the South African context, where healthcare systems often face resource constraints and a growing burden of non-communicable diseases such as stroke and neurodegenerative conditions (Said et al., 2023).

These findings about nursing students’ undergraduate training and nurses’ knowledge and confidence in dysphagia highlight the need for educational interventions to improve future nurses’ competency (Matlhaba, 2025). Additionally, they underscore the pressing need for more research into nursing students’ educational experiences related to dysphagia management, especially in LMICs. The focus on LMICs is based on research by Pillay and Pierpoint (2020), which found that in high-income countries, nurses partake in screening and training programmes to prepare and improve their practices in dysphagia, something that is yet to be implemented in LMICs (Robbertse & De Beer, 2020c). By focusing on a low- to middle-income setting, the study also seeks to contribute to broader efforts in strengthening dysphagia education and interdisciplinary practice across healthcare systems. Filling these gaps is essential for enhancing patient outcomes and reducing potential legal issues arising from insufficient care (Jones & Porterfield, 2020b).

This study aimed to investigate the educational experiences and challenges faced by nursing students in managing dysphagia, explore the supportive strategies they employ and make recommendations to enhance dysphagia management education for future nursing students. To achieve this aim, the study had three specific objectives: (1) to examine the challenges nursing students encounter in dysphagia management, (2) to identify the supportive strategies students use to address these challenges and (3) to develop evidence-informed recommendations for improving dysphagia management education based on students’ experiences and strategies. The research was guided by an interprofessional collaboration model and adult learning theory, both of which emphasise the value of experiential learning and team-based approaches in preparing healthcare professionals for clinical practice (Brooks et al., 2016; Cichero et al., 2017a; Dondorf et al., 2015b).

## Research methods and design

### Setting

The research was conducted at a higher education institution in the Eastern Cape province, South Africa. The institution, recognised as historically disadvantaged, hosts an estimated 13 000 students, most from previously disadvantaged backgrounds. The nursing department at this institution began in 1984 as a joint agreement between the university and the Department of Health to educate and train nurses to increase the number of nurses to serve the homelands of Ciskei, as the lack thereof was extreme (Evertse, 1997). The setting is significant because it reflects a typical academic and clinical training environment where nursing students are prepared for the complex realities of healthcare delivery in a resource-constrained environment.

### Study design

This study utilised a descriptive phenomenological qualitative design in which, in 2024, 12 registered final-year nursing students at the University of Fort Hare in the Eastern Cape, who met the study’s inclusion criteria, were recruited out of 27 students through purposive sampling to participate in semi-structured interviews about their experiences with dysphagia management (Fugard & Potts, 2015). Recruitment involved visiting the Nursing Department at the East London campus and personally inviting students to participate through word of mouth. The study explored experiences of final-year nursing students in the university who have received theoretical knowledge on dysphagia and were currently gaining clinical experience at the time in clinical settings such as acute stroke units, aged care facilities and neurology wards, where they are likely to encounter patients with dysphagia.

### Inclusion and exclusion criteria

To ensure that the study participants were appropriate and could provide reliable and meaningful data relevant to the research question, specific inclusion and exclusion criteria were applied. The inclusion criteria required participants to be students registered at the University of Fort Hare in the 2024 academic year, pursuing a nursing degree, in their final year of study, and having encountered cases of dysphagia during their clinical practice. Conversely, participants were excluded if they were not currently registered at the University of Fort Hare, not enrolled in 2024, not pursuing a nursing degree, not in their final year of study or lacking clinical experience with dysphagia.

### Data collection

Data were gathered through semi-structured in-person interviews conducted in English, which lasted approximately 35 min – 45 min using interview guides developed by the researchers following an extensive literature search (DeJonckheere & Vaughn, 2019; Ruslin et al., 2022). A questionnaire (Appendix 1) with 10 open-ended questions served as the primary data collection tool, focused on confidence in theoretical knowledge, practical skills and formal training in dysphagia management, the number of dysphagic patients encountered, challenges faced and strategies used when caring for patients with dysphagia, as well as understanding their knowledge of the role of SLPs in dysphagia management. To ensure face and content validity, the interview guide was reviewed by two experienced speech therapists; no changes were made after the review. A pilot study was conducted with five nursing students. During this phase, researchers posed questions outlined in the interview guide to assess their appropriateness, which were conducted before data collection for the main study. The sessions were audio-recorded to ensure accuracy and to enable the interviewer to focus on active listening and building rapport rather than extensive note-taking. Thereafter, researchers consulted with the research supervisor to make necessary adjustments, as some questions were rephrased. Data from the pilot study were excluded from the primary analysis to maintain rigour. The interviews were conducted by researchers at the East London University Campus.

### Data analysis

To ensure accuracy, interview recordings were transcribed verbatim and carefully checked against the audio files. The transcripts were anonymised by replacing names with participant numbers to maintain confidentiality. The data were analysed using thematic analysis following Braun and Clarke’s six-step framework: familiarisation with the data, generating initial codes, searching for themes, reviewing themes, defining and naming themes and producing the final report (Braun & Clarke, 2006; Clarke & Braun, 2013). Coding was developed collaboratively by the research team, and ATLAS.ti was used to organise, code and explore patterns in the data while ensuring that interpretive decisions were guided by the researchers rather than the software alone (Soratto et al., 2020).

### Trustworthiness of the research

Validity and reliability in qualitative research mean the extent to which the data are trustworthy and thus can be defended when challenged (Noble & Smith, 2015). As such, the data were returned to the participants of this study so that they could verify the accuracy of the recorded information (Birt et al., 2016) to ensure credibility. Transferability in this research was ensured by a thick description (Tayade & Lattie, 2021). Thick description entails providing a comprehensive narrative of field experiences, wherein the researcher explicitly outlines the cultural and social relationship patterns and contextualises those (Younas et al., 2023). In this research, dependability was ensured by an audit inquiry. An inquiry audit entails an external researcher, independent of both the data collection and analysis, reviewing the procedures of data collection, data analysis and the research study’s outcomes (De Kleijn & Van Leeuwen, 2018; McLeod, 2024.). In this research, the research supervisor was the one acting as an external researcher to review the research process. Confirmability in this research was ensured by an audit trail. An audit trail comprises clear and transparent documentation of the research process, starting from the initiation of the project through to the formulation and presentation of findings (De Kleijn & Van Leeuwen, 2018). The above criteria ensure that the results are trustworthy and thus valid and reliable, without any form of bias, thereby enhancing the overall integrity of the research findings.

### Ethical considerations

Ethical approval for this study was obtained from the University of Fort Hare Health Research Ethics Committee (UFH HREC) (approval number: Ref #2024-09-18 HRECLVN11). Permission to conduct the study was also granted by the Head of the Nursing Department and relevant institutional gatekeepers. Written informed consent, including consent for audio recording, was obtained from all participants prior to data collection. To ensure confidentiality, participants were assigned identification numbers, and no personal identifying information was recorded.

## Results

Twelve final-year nursing students (*n* = 12), comprising six male and six female students aged 22–34 years, were interviewed. All participants reported a lack of confidence in their ability to manage dysphagic patients. The interviews revealed a range of challenges, as well as facilitators, strategies employed and recommendations for improvement. In accordance with the aim and objectives of the study, the following four main themes were identified through thematic analysis: (1) confidence and knowledge, (2) clinical exposure, (3) current dysphagia management practices and (4) development of clinical skills.

### Theme 1: Confidence and knowledge

The first theme of confidence and knowledge emerged in relation to participants’ experiences with dysphagia management, encompassing theoretical knowledge, practical skills and formal training (Table 1). Skills were reported to be developed through hands-on experiences and guidance from senior nurses, though one participant emphasised the challenge of not having access to speech, while 11 participants acknowledged feeling underprepared to manage patients with dysphagia:

‘I’m not confident enough with theory. So I can say I don’t have any confidence for the theoretical side.’ (Participant 4, male, 26 years old)

**TABLE 1:** Nursing students’ experiences with dysphagia education and practice.

Sub-theme	Summary	Sample quote
Theoretical knowledge	Dysphagia was covered briefly, perceived as superficial	‘I’m not confident enough with theory …’ (Participant 4, male, 26 years old) ‘We did touch on dysphagia in class, but it was very superficial … I wouldn’t say I feel knowledgeable about it.’ (Participant 11, male, 25 years old)
Practical skills	Limited hands-on opportunities affected readiness	‘I’m still not 100% confident because I have to feed the patient, I feed the patient through the tubes [*nasogastric and orogastric tubes*]. I have never had a patient to feed.’ (Participant 6, male, 26 years old) ‘It depends on the wards and the conditions of the patients, because with the medical wards I do see people there with the procedure as well as the, the, on the neonatal unit side, which we call the nursery. So more especially the preterm babies. So they struggle to suck as well as the small babies. So the management on that side it’s if the patient is not swallowing. Well, so the, the orders might be the insertion of the nasal, gastric or soft food and just help the patient by feeding the patient.’ (Participant 4, male, 26 years old)
Formal training	Absence of dedicated dysphagia training	‘… I wouldn’t really say formal training … not like we have learned about the condition and how its managed, we have learnt about neurological problems and how some of them could lead to dysphagia, so dysphagia was just a symptom of the neurological disorder.’ (Participant 2, male, 45 years old) ‘Training, I can’t call it training because it was all about seeing how to take care of a patient who cannot swallow, not that it’s a training. It’s not a training.’ (Participant 3, male, 25 years old)

This also reflected a strong awareness of the complexity of care and a motivation to improve. Six Participants described their current theoretical knowledge as ranging from low to moderate:

‘On a scale of 10, I would give it 4, because I’m a nurse most of the patient that we deal with in medical wards and casualty and ICU [*intensive care unit*], they usually have swallowing difficulties.’ (Participant 9, female, 28 years old)

often shaped by brief and lecture-based instruction. However, this recognition highlighted a proactive desire for deeper learning. Nine participants expressed the need for dedicated modules, workshops and more comprehensive training that was consistent across participants, pointing to a constructive opportunity for curriculum development:

‘It must be introduced in our curriculum and also practical training can be beneficial so that we can understand dysphagia management better.’ (Participant 6, male, 26 years old)

Overall, while gaps were identified, the findings underscore a clear commitment among learners to enhance their competencies and advocate for improved educational resources in dysphagia management.

In terms of practical skills, participants expressed varied levels of confidence and competence based on their experiences during clinical placements. Despite limited theoretical training, five participants felt confident in performing routine procedures, such as feeding through nasogastric (NG) tubes:

‘I’ll say that many patients that have difficulties in swallowing, we insert the NGT [*nasogastric tube*] for feeding.’ (Participant 1, male, 27 years)

because these tasks were frequently encountered and practised in the wards. Practical therapists regularly. Similarly, participant 5 reported confidence in practical aspects like spoon-feeding patients but admitted their confidence was uncertain because of the lack of theoretical knowledge:

‘Well, because of it, it is not something I think is difficult to do. Like it’s like helping someone who can’t swallow. Maybe you help them taking out the spoon and like, spoon feeding them and all that, so it’s not something that I would say it writing it about my confidence as such. But I can see that maybe I am helping those people because they have to take treatment. So before you take treatment, you have to eat. So yeah, maybe I am confident. Maybe I am not. I don’t know for sure where.’ (Participant 5, female, 26 years old)

This limited theoretical exposure contributed to a lack of confidence in recognising and managing swallowing difficulties. Moreover, participants highlighted a scarcity of hands-on training opportunities, further impeding their ability to translate knowledge into clinical competence.

### Theme 2: Clinical exposure

Under this theme, seven participants reported that their clinical exposure to dysphagia varied considerably depending on the ward or placement (Table 2):

‘It depends on the wards and the conditions of the patients, because with the medical wards I do see people there with the procedure as well as the, the, on the neonatal unit side, which we call the nursery. So more especially the preterm babies. So they struggle to suck as well as, the small babies. So the management on that side it’s if the, the patient is not swallowing. Well, so the, the orders might be the insertion of the nasal, gastric or soft food and just help the patient to by feeding the patient.’ (Participant 3, male, 25 years old)

**TABLE 2:** Patient encounters, challenges, and support in dysphagia management.

Sub-theme	Summary	Sample quote
Patient encounters	Exposure varied widely depending on the clinical ward	‘It depends on the wards and the conditions of the patients, because with the medical wards I do see people there with the procedure as well as the, the, on the neonatal unit side, which we call the nursery. So more especially the preterm babies. So they struggle to suck as well as the small babies. So the management on that side it’s if the patient is not swallowing.’ (Participant 4, male, 26 years old) ‘I usually encounter them in the medical ward whereby you find that maybe there are people who are ill and they cannot feed themselves …’ (Participant 5, female, 26 years old) ‘I usually see them in ICU [*intensive care unit*] and medical wards, in ICU [*intensive care unit*] I can say I see 90% and they are few in other wards. In nicu there are also many babies with feeding tubes.’ (Participant 6, male, 26 years old)
Challenges encountered	Difficulty recognising dysphagia signs and interventions	‘I can say for myself the challenges that I face is you just give them food, others cannot even feed themselves and then you don’t know what to do when the patient is having a problem or coughing whilst you are feeding them or choking you are just told that the patient can’t feed themselves and you have to feed the patient and during the process of feeding and then the patient chocks you just stop or give them water.’ (Participant 8, female, 27 years old) ‘For me, since I was in second year at that time, I didn’t know how to take care of that patient.’ (Participant 12, female, 25 years old)
Lack of support	Insufficient guidance on dysphagia management	‘So as well as it also depends on the or as well the speech therapies. They do not come to the wards … We do not get training from a speech therapist we like to see, to observe when the speech therapist is with the patient.’ (Participant 4, male, 26 years old)

Those assigned to stroke units, medical wards and the intensive care unit (ICU) encountered patients with swallowing difficulties more frequently:

‘We do in medical wards. Maybe stroke patients and surgical wards with patients having problems with the throat. I have encountered some.’ (Participant 2, male, 45 years old)

In contrast, others, like the maternity ward, had minimal exposure. This inconsistency led to uneven opportunities for learning and skill development. In terms of challenges encountered, participants also described challenges in identifying early signs of dysphagia and uncertainty regarding intervention steps. Aspiration during feeding was reported to be a significant concern among the participants because they do not know what to do when a patient coughs while being fed:

‘I can say for myself the challenges that I face is you just give them food, others cannot even feed themselves and then you don’t know what to do when the patient is having a problem or coughing whilst you are feeding them or chocking you are just told that the patient can’t feed themselves and you have to feed the patient and during the process of feeding and then the patient chocks you just stop or give them water.’ (Participant 2, male, 45 years old)

Lastly, three participants noted limited supervision or mentorship related to dysphagia management during clinical placements, especially from speech therapists:

‘And it’s that I, as I’ve said on the on the first question that we are not theoretically related. So we do things that we’ve we have been trained by the sisters like the insertion of the nasal gastric. So as well as it also depends on the or as well the speech therapies. They do not come more often to the wards. And when they arrive to the wards trying to help patients. So it’s time consuming because at that time we as nurses might be doing something else. So I can say it is a challenge because we do not get a training from a speech therapist who likes to see, to observe when the speech therapist is within the patient. So that’s the challenge.’ (Participant 4, male, 26 years old)

This noted gap in support contributed to their feelings of uncertainty and reduced confidence in practice. Table 2 provides an overview of this theme.

### Theme 3: Current dysphagia management practices

This theme encompasses sub-themes such as current strategies, role of SLPs and resource needs (Table 3). Twelve participants reported various strategies utilised in their clinical practice to support patients with dysphagia. Common approaches included (1) inserting a nasogastric tube:

‘Normally for us, we insert tube and monitor if it’s in the right place, etc.’ (Participant 11, male, 25 years old)

**TABLE 3:** Current strategies and the role of speech therapists in dysphagia management.

Sub-theme	Summary	Sample quote
Current strategies	Use of nasogastric (NG) for feeding patients with dysphagia	‘I use nasogastric tubes.’ (Participant 1, male, 27 years old) ‘We use NG-tubes [*nasogastric tubes*], to ensure that they do receive nutrition.’ (Participant 3, male, 25 years old) ‘I’ll say that many patients that have difficulties in swallowing, we insert the NGT [*nasogastric tube*] for’. (Participant 12, female, 25 years old)
Role of speech therapists	Acknowledged importance but limited knowledge about their role	‘I think it’s very very important, I didn’t know this, I thought speech therapy only focused on like speaking, I never thought that it would focus on swallowing until I was at the nursery wards, at the nursery ward there were children, pre term babies who could not suck and not swallow and the speech therapist were there, and I was like what is a speech therapist really doing here, and I think it is really important and like I have said as nurses we don’t really have hands on management on dysphagia so it is important for the speech and language therapist to be there.’ (Participant 2, male, 45 years old) ‘I usually see the speech therapist when they come to the ward. So trying to train the patient on how to, on how to swallow. And they are easily to they are easily to observe if the patient is having difficulties on the swallowing. So I might say their role is very, very, very important because they are so observant in the swallowing process.’ (Participant 4, male, 26 years old)

and (2) the correct positioning of patients:

‘I have developed like changing the positions and also lifting the bed and we don’t even have the angle on how you lift the bed.’ (Participant 8, female, 27 years old)

Of noteworthy is also the observed consistency in dysphagia management across clinical settings. Regarding the role of the SLP, while participants I acknowledged the vital role of SLPs, they frequently expressed uncertainty about when and how to refer patients:

‘Hence [*I*] am saying, I have seen most patients with that kind of difficulty being referred to speech therapists. And I’ve seen them try to work with them. For me the ones I have seen being referred, it depends on the severity of the condition, I’ve seen quiet an improvement. I remember a guy at refer being referred to speech and after 2 weeks you see the improvement in his swallowing reflexes.’ (Participant 10, female, 27 years old)

### Theme 4: Development of clinical skills

A recurring theme among participants was the desire for structured learning opportunities to enhance their clinical skills in dysphagia management (Table 4). Five participants expressed interest in simulation-based training to practice assessment and intervention techniques in a safe environment before real patient encounters:

‘Resources and support? I guess, since I don’t have much knowledge about it, I think the very best think would be in-service training, since maybe me as a student I haven’t really been hands on in management of dysphagia, I would have in-service training on how to be better involved in managing it and not just doing nasogastric feeds as ordered maybe also me being able to see that this patient has a better swallowing reflex now.’ (Participant 1, male, 27 years)

**TABLE 4:** Competence development and the need for clear dysphagia protocols.

Sub-theme	Summary	Sample quote
Competence improvement	Importance of dysphagia training	‘By getting in-service training about dysphagia.’ (Participant 1, male, 27 years old) ‘I think we should have in service training whereby we are taught more about dysphagia and in service training with speech therapist on how to manage a patient with dysphagia, that would be a bit help when collaborating with speech and nursing.’ (Participant 7, female, 25 years old)
Management protocols	Need for clear, accessible protocols	‘The protocols that are applied on the management of this dysphagia. So I might say on, on the clinical settings or starting from the scope of practice. So it depends on the scope of practice because you cannot say a first year student or you cannot allocate a first year student to insect tube, or a staff nurse or nursing assistant to insert the tube. So it depends on the scope of practice. So those categories that I have mentioned. So they can only assist on the on the fitting. So by positioning the patient and try to feed the patient. But if that patient cannot swallow so it leads to the patient to be inserted or to be inserted in the tube. So those are the ways of protocols that are used in the clinical settings.’ (Participant 4, male, 26 years old)

Factors such as collaboration between the speech therapy and nursing departments were brought up by participants in efforts to improve the management of dysphagia in patients (Bardien et al. 2020):

‘I think we should have in-service training whereby we are taught more about dysphagia and in-service training with speech therapists on how to manage a patient with dysphagia. That would help when collaborating with speech and nursing.’ (Participant 7, female, 25 years old)

Participants also emphasised the need for accessible, clear management protocols to guide clinical decision-making:

‘The protocols that are applied on the management of this dysphagia. So I might say in [*sic*] clinical settings or starting from the scope of practice.’ (Participant 4, male, 26 years old)

Such resources were seen as essential to improve confidence, ensure consistency and promote evidence-based practice among nursing professionals.

## Discussion

This study highlights a critical gap in final-year nursing students’ preparation for dysphagia management. Across interviews, a consistent picture emerged: students recognised the importance of safe dysphagia care and valued collaboration with SLPs, yet they reported insufficient theoretical grounding, uneven clinical exposure and low confidence in recognising and managing swallowing difficulties. These findings have important implications for patient care, nursing education and professional theory.

### Significance of the findings

The most striking finding is the mismatch between students’ awareness of the seriousness of dysphagia and their limited competence to manage it. Although many had performed routine tasks such as nasogastric feeding, they lacked a deeper understanding of dysphagia assessment and intervention. This combination of low theoretical knowledge and limited mentorship echoes global evidence that underprepared nurses are at risk of missing early signs of swallowing disorders (Hazelwood et al., 2025; Jeon et al., 2024; Jones & Porterfield, 2020; Olímpio et al., 2024). In settings where clinical supervision varies widely, such gaps can have serious consequences.

### Implications for patient safety and clinical practice

Dysphagia is a potentially life-threatening condition: malnutrition, dehydration, aspiration pneumonia and mortality are well-documented risks when management is inadequate (Dziewas et al., 2021; Eltringham et al., 2019). Students described uncertainty when patients coughed or choked during feeding and reported inconsistent guidance from mentors and SLPs. Without clear protocols or reliable supervision, bedside practice becomes variable and may raise ethical and medico-legal concerns. These findings, therefore, call for health-care institutions to implement accessible, standardised protocols and ensure that nurses receive ongoing support from experienced clinicians.

### Implications for training and education

From an educational perspective, the study points to the need for a more integrated and clinically relevant approach to dysphagia training. Participants advocated simulation-based learning and structured opportunities to observe and practice under expert supervision. Evidence supports that simulation and guided practice strengthen competence and confidence in dysphagia care (Adams et al., 2024; Carless-Kane & Nowell, 2023a; Daneshfar & Karimi Moonaghi, 2025a; Hazelwood et al., 2025b). Early, repeated clinical exposure, reinforced by feedback, would enable students to translate theory into safe practice.

Interprofessional education is equally important (Bloom et al., 2022). Students’ uncertainty about when and how to refer patients to SLPs highlights the need for joint learning activities. Collaborative training with speech therapy departments would help nursing students understand SLP roles and foster effective referral pathways (Dondorf et al., 2015a; Khalili et al., 2022; Lal et al., 2024; Wilkinson et al., 2021). Nursing curricula should therefore embed dysphagia management as a core component and create formal opportunities for interprofessional collaboration.

### Contribution to theory

These findings reinforce the central tenet of experiential learning theory that competence develops through structured observation, simulation and guided participation (Matlhaba, 2024). When students actively engage with real or simulated patients and receive timely feedback, they integrate theoretical knowledge with clinical judgement, building both confidence and professional identity. The results thus extend evidence that experiential learning approaches are particularly valuable in resource-limited contexts where clinical exposure is often inconsistent.

### Future directions

Future research should evaluate the long-term impact of simulation-based and interprofessional training on both student competence and patient outcomes. Studies in LMICs are especially needed to examine how such interventions can be adapted to settings with variable staffing and limited access to SLPs (Kathard & Pillay, 2013).

## Conclusion and recommendations

The findings of this study, conducted with nursing students at Eastern Cape University, highlight the need to enhance dysphagia education and clinical practice within this specific context. Incorporating comprehensive, evidence-based training that combines theoretical instruction with practical experiences, such as simulation and structured clinical exposure, may improve student competence and confidence (Carless-Kane & Nowell, 2023b; Daneshfar & Karimi Moonaghi, 2025b; Hazelwood et al., 2025b). Structured interprofessional education with SLPs, standardised clinical protocols and mentorship can further support effective learning and safe patient care (Jones & Porterfield, 2020c; Khalili et al., 2022; Robbertse & De Beer, 2020b). However, it is important to acknowledge that these findings are based on the experiences of only 12 nursing students, which limits the extent to which the results can be generalised. While these recommendations are based on students’ experiences at Eastern Cape University, similar strategies may be adapted in other nursing programmes with careful consideration of local contexts, resources and student populations (Di Giorgio et al., 2020; Jeon et al., 2024; Kaylor & Singh, 2023). Implementing these approaches can strengthen students’ preparedness, foster collaborative practice and improve the quality and safety of dysphagia care without assuming universal generalisability.

## Appendix 1: Research questionnaire

### Questionnaire

1. How confident do you feel in your theoretical knowledge of dysphagia management?
2. How confident do you feel in your practical skills related to dysphagia management?
3. Have you received any formal training in dysphagia management during your nursing education?
4. How often do you encounter patients with dysphagia in your clinical placements?
5. What are the most common challenges you face when caring for patients with dysphagia?
6. What strategies do you currently use to support patients with dysphagia in your clinical practice?
7. How do you perceive the role of speech and language pathologists in dysphagia management?
8. What resources or support do you feel would be most beneficial in improving your dysphagia management skills?
9. How do you think your clinical competence in dysphagia management could be further developed?
10. What are your perceptions of the current dysphagia management protocols in the healthcare facilities where you have completed clinical placements?
